# Evaluation and Optimization of Walkability of Children’s School Travel Road for Accessibility and Safety Improvement

**DOI:** 10.3390/ijerph19010071

**Published:** 2021-12-22

**Authors:** Jia Zhao, Wei Su, Jiancheng Luo, Jin Zuo

**Affiliations:** 1 School of Architecture, Tianjin University, Tianjin 300072, China; ziyujiuyou@163.com (J.Z.); tosuwei@163.com (W.S.); 2 Beijing Municipal Institute of City Planning & Design, Beijing 100045, China; 3 Tianjin University Urban Planning and Design Institute Co., Ltd., Tianjin 300072, China; 4 College of Resources and Environment, University of Chinese Academy of Sciences, Beijing 100049, China; luojc@radi.ac.cn; 5 Aerospace Information Research Institute, Chinese Academy of Sciences, Beijing 100101, China; 6 Tianjin Laboratory of Creative Urban Design, Tianjin University, Tianjin 300072, China

**Keywords:** children, pedestrian school travel road, accessibility, safety, optimization verification

## Abstract

(1) Background: In the context of a children friendly city, accessibility and safety are the basic needs of children’s pedestrian school travel. This study proposes a comprehensive evaluation method of pedestrian accessibility and safety for children’s school travel. (2) Methods: Firstly, the school travel network was constructed by simulating the path of children walking to school. Secondly, from the meso and micro dimensions, the impact factors of pedestrian accessibility and safety were combed out, and an evaluation index system was constructed. Finally, pedestrian accessibility and safety were evaluated based on the Space Syntax analysis and ArcGIS spatial analysis, and the results were superimposed and spatially differentiated. The new evaluation method was tested in the Jintang Road area in Hedong District, Tianjin, China. (3) Results: The pedestrian accessibility and safety of children’s school travel road in the study area needed to be improved. It was found that the main impact factors were the effective walking width, the spatial connectivity, the visual integration, the obstruction of pedestrian safety, the completeness of crossing facilities and the influence of traffic flow and put forward optimization strategies. After optimized simulation verification, the overall improvement was achieved. (4) Conclusion: The evaluation method is helpful to calculate the pedestrian accessibility and safety of children’s school travel, and help decision makers determine the design and management strategies of child-friendly streets.

## 1. Introduction

With the development and transformation of urban planning from incremental construction to focus on people-oriented inventory renewal and refined governance, the construction of Children Friendly City has become an important direction for urban development. The United Nations series of advocacy documents have repeatedly emphasized the importance of the community as the basic spatial unit of children’s daily activities in building Children Friendly Cities [1,2]. As an important place for children in the community living circle to contact society and nature, the school travel road is an important place for children to promote the health and safety of active school travel, which can provide important support for the construction of Children Friendly Community [3].

So far, the research and practice of children’s school travel road in the world has been relatively early. The construction of active school travel roads had become an important part of the construction of Children Friendly Cities and Communities in many countries, such as the Safe Routes to School program in the United States, the Walking School Bus program in New Zealand and the gelbe Fußabdrücke program in Germany, which had carried out a lot of practical work and achieved remarkable results [4,5,6]. Domestic research was relatively late. At present, some achievements have been made in the aspects of children’s school travel characteristics, restrictive factors and the impact of built and non-built environments [7,8,9]. In terms of the walkability evaluation methods of school travel roads, most of them were separately studied for their walkability or safety, and few combined the two indicators as comprehensive indicators for unified research [10,11,12,13]. The research path was mostly “Status Evaluation-Optimization Strategy”, lacking a feedback mechanism for the effectiveness, rationality and operability of the optimization scheme. In terms of planning convergence, the relevant design provisions of school travel road in China’s current specifications and guidelines were relatively scattered, and there was less special research on children’s pedestrian school travel, which was insufficient to link with the existing planning [14,15,16,17].

Through the relevant research on children’s behavior patterns and cognitive psychology [18], it was found that school-age children’s school travel activities had fixed time, place and action path. In the process of walking through school, children had such behavioral characteristics as small range of activities [19], narrow vision and slow pace [20], self-centered and weak sense of rules [21,22] and frequent interaction with facilities [23]. Additionally, there are physiological constraints such as short travel distance and slow speed, as well as safety constraints such as high demand for safety and need to accompany [9]. Combining with Maslow’s hierarchy of needs theory [24] and the Mitra’s behavior model of school travel (BMST) [25], school-age children have accessibility, safety, and comfort requirements for pedestrian school travel [26]. Among them, accessibility and safety are rigid requirements, which are the controlling elements of the evaluation of children’s walkability on the school travel road, and should be given priority.

To sum up, this study carries out the evaluation and optimization of the walkability of children’s school travel roads from the perspective of community life circle, focuses on solving the problems of accessibility and safety requirements. On the one hand, this study discusses how to comprehensively consider the children’s walkability and safety needs of school travel roads and how to carry out comprehensive evaluation of control indicators. On the other hand, it discusses how to comprehensively consider the children’s walkability and safety needs of school travel roads, and construct a research route with strong operability.

## 2. Materials and Methods

### 2.1. Research Object

According to different classification standards, the “Convention on the Rights of the Child” defines a child as anyone under the age of 18 [27].”Pediatrics” divides the age of children into 7 stages: before the age of three years, they are fetus, newborn, infant and early childhood, from 3 to 6~7 years old is pre-school, from 6~7 to 12 years old is school-age, and then adolescence [28]. In this study, school-age children aged from 6 to 12 years old at the primary education stage were selected as the main research objects. Children in this age group have the closest contact with school travel road and have a strong demand for walking activities.

“School Travel” is one of the main daily activities of school-age children. In Japan, the only way for primary and middle school students to go to school is called “School Travel Road” in order to ensure the safety of children’s school travel and maintain a good education environment [29]. In this study, the “school travel road” is defined as the road between the residential area and school for school-age children, including the urban space composed of buildings, structures and facilities adjacent to the road.

Accessibility and safety are the rigid needs of children’s school travel. Combined with BMST model [25], children’s activities on the street are mostly associated with travel activities. There is a relationship between different spatial elements and needs of school travel road, and children are affected by physiological constraints and safety constraints. Therefore, in different degrees of meso and micro, children’s different cognitive and perceptual abilities when using the road environment are the basic elements to evaluate accessibility and safety. Accessible and safe walking network and walking space are the rigid requirements to ensure children’s walking accessibility.

### 2.2. Study Area

**Figure 1:**
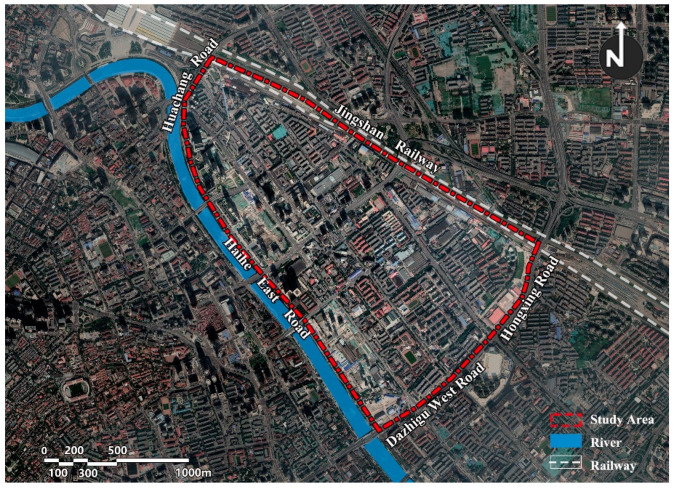
Study area.

This study selected Tianjin Jintang Road area as a typical research area with an area of about 3.34 km^2^. It is located in the central area of the city, on the east bank of the Haihe River, adjacent to Tianjin Railway Station. It is a typical high-density area with diverse residential areas (Figure 1). The research area includes two primary schools and three campuses. The surrounding road network is dense, the road space is narrow, and the flow of traffic and people is large. Combined with the principle of “nearby enrollment” [30,31] and the provisions of China’s “Urban Residential Area Planning and Design Standard” (GB50180-2018) that primary schools should be equipped with public service facilities for 10-min community living circle [32], the general distance of school-age children’s school travel in this study was ten minutes walking distance.

### 2.3. Research Methods

#### 2.3.1. Data Base and Network Construction

In this study, the spatial, time and attribute data were obtained through field survey, questionnaire survey, mapping and network data crawling. Among them: spatial data included the spatial information of land use, buildings, supporting facilities and roads, which are mainly obtained through Open Street Map, Baidu Map Place API and Baidu POI. Time data included the timing of construction (current, under construction, or planning), as well as the morning peak (7:20–7:50) and evening peak (15:00–16:00) of children’s school travel, which are obtained through on-site photos, video sampling and Baidu Map—Road Congestion Map. The attribute data included the attribute information of land use, buildings, supporting facilities and road data, as well as children’s walking behavior data and street view data, which are obtained through Baidu Map Place API, Baidu POI, on-site photos and video sampling.

**Figure 2:**
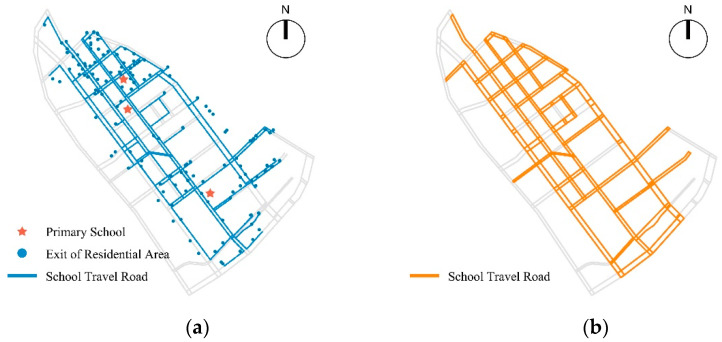
(**a**) Analysis of the shortest distance of children’s pedestrian school travel; (**b**) the basic network of children’s pedestrian school travel road.

Finally, it integrated multi-source data through data format conversion and unification of spatial references, and used ArcGIS shortest path analysis to calculate the shortest path from each residential entrance to the primary school entrance. Then, it combined with the actual research situation to screen children’s school travel roads and build a school travel road network (Figure 2).

#### 2.3.2. Index System Construction

Based on the principles of accessibility and safety, combined with the relevant literature, evaluation tools and related indicators, the rationality of indicators and the difficulty of data acquisition, as well as the actual needs and behavior characteristics of children’s school travel, this study finally summarized 10 core factors that affected the accessibility and safety of walking from the meso and micro dimensions. The index system was constructed from target level, criterion level and index level (Table 1).

#### 2.3.3. Evaluation Model Construction

The evaluation of pedestrian accessibility and safety of children’s school travel road was mainly carried out from two levels: graded evaluation and comprehensive evaluation. The graded evaluation part included pedestrian accessibility evaluation based on the segment analysis and ArcGIS spatial analysis, and pedestrian safety evaluation based on visibility graph analysis and street view data analysis. The weight of each index (Table 2 and Table 3) and grading evaluation results were calculated by entropy weight method; the comprehensive evaluation was based on the superposition analysis and classification evaluation, analyzed its spatial differentiation characteristics, and explored the influence mechanism of spatial factors on children’s school travel road.

### 2.4. Technical Route

**Figure 3:**
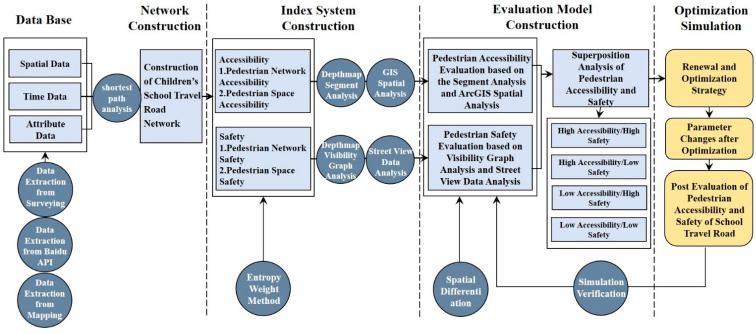
Technical route.

The study took “Data Base and Network Construction-Index System Construction-Evaluation Model Construction-Post Optimization Evaluation” as the route to carry out the evaluation and optimization of the walkability of children’s school travel roads based on accessibility and safety requirements. In this way, the closed logical loop of “Status Evaluation-Optimization Strategy-Post Optimization Evaluation” was formed, which provided technical support for the regeneration and optimization of the school travel road (Figure 3).

## 3. Results and Discussion

### 3.1. Analysis of Children’s School Travel Needs and Problems Based on Field Investigation

In the field investigation part, taking the height of children (1.3 m) as the point of view, street view photos and videos were taken on the road during the school travel period. The photos should include space elements such as sidewalks, intersections, street facilities, and parking spaces, as well as information on children’s behavior and activities.

In the questionnaire survey part, questionnaire interviews were conducted with primary school children and their parents in the study area during the school hours (15:00–16:00), focusing on their actual needs for school travel roads and their subjective feelings on various factors affecting the accessibility and safety of school travel.

Among them, 102 school-age children and their parents answered the questionnaire, and 98 valid questionnaires were obtained. This paper summarizes the current situation and problems of school travel for school-age children in the study area, so as to provide a basis for subsequent optimization design and renovation.

**Figure 4:**
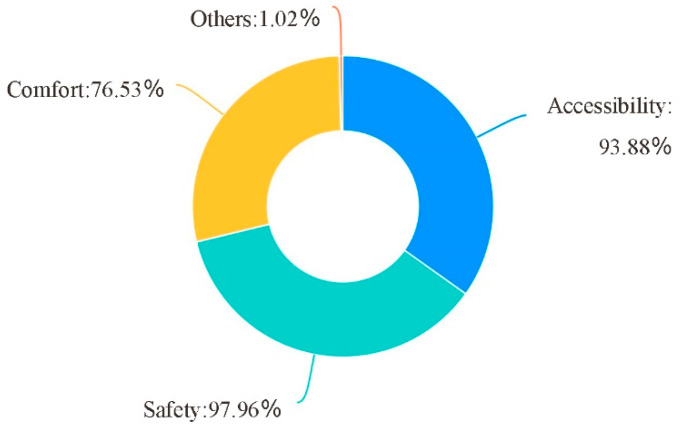
School age children’s demand for school travel road.

Children’s school travel activities are mainly divided into walking, staying and playing activities (Table 4). Walking is the main behavior of school travel. Whether it is walking through, crossing the street or chasing and playing, accessibility and safety are the most basic needs. According to the survey results, 97.96% of the people had the demand for the safety of the pedestrian school travel road, 93.88% had the demand for the accessibility, and the accessibility and safety were the rigid demand of the pedestrian school travel (Figure 4).

**Figure 5:**
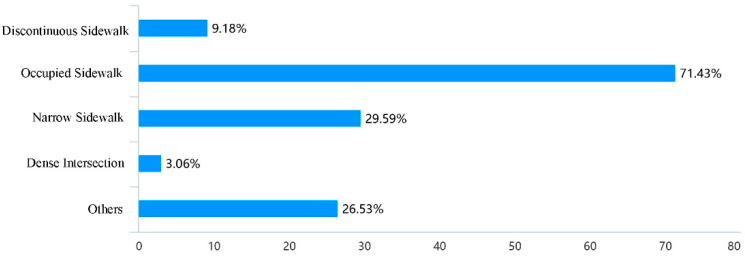
Factors causing pedestrian inaccessibility of the current school travel road.

**Figure 6:**
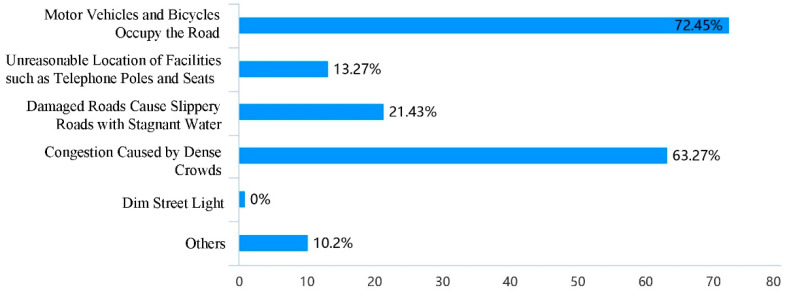
Factors causing unsafe walking of the current school travel road.

**Figure 7:**
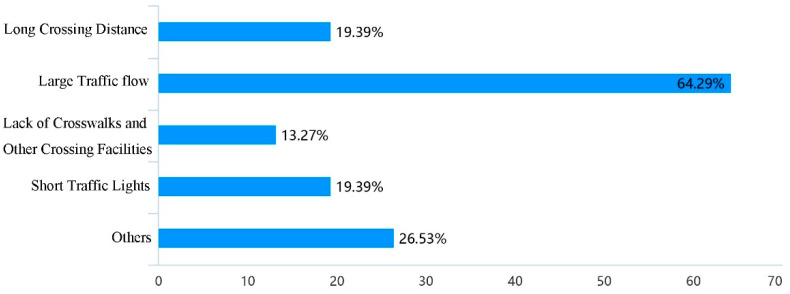
Factors causing unsafe crossing of the current school travel road.

**Figure 8:**
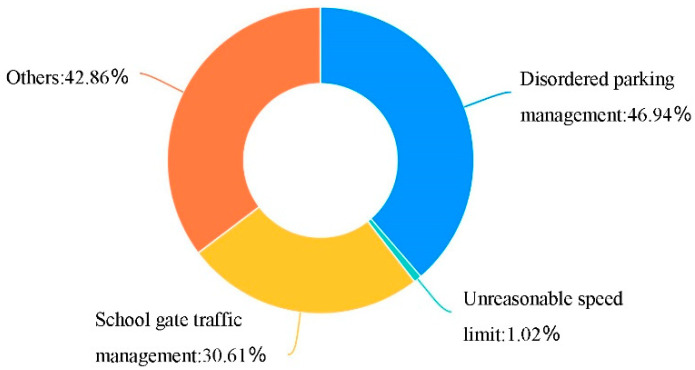
The lack of management of the current school travel road.

Based on the evaluation of the use of school travel road, through the analysis of the influencing factors of pedestrian accessibility, it was found that the factors such as the discontinuity, occupation and narrow of the sidewalk could cause the obstruction of school travel road, among which the narrow of the sidewalk was the most important reason (Figure 5); Through the analysis of the influencing factors of pedestrian safety, it was found that parking of motor vehicles and bicycles and facilities obstacles on the sidewalk were the important reasons for the unsafety of the current school travel road (Figure 6); At the same time, the long crossing distance, large traffic flow, lack of crosswalks and other crossing facilities have caused unsafe impact on pedestrian crossing (Figure 7). In addition, 46.94% of the people thought that the current parking management was chaotic, while 30.61% thought that the school gate traffic management was insufficient, and the police should be strengthened in the management of the school travel road (Figure 8).

To sum up, based on the actual feelings of school-age children and their parents, combined with the influencing factors of the current situation of pedestrian accessibility and safety in the study area, it was concluded that the optimization of the school travel road should focus on solving the following problems: occupied walking space; lack of street crossing facilities; conflict between people and vehicles at the school gate; lack of current system guarantee, etc.

### 3.2. Pedestrian Accessibility Evaluation Based on the Segment Analysis and ArcGIS Spatial Analysis

**Figure 9:**
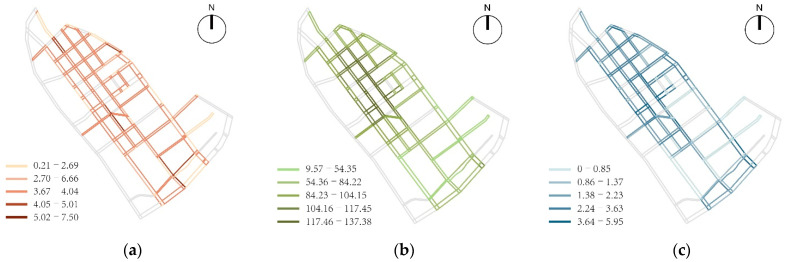
The analysis of pedestrian network accessibility: (**a**) Angular Connectivity; (**b**) Integration R600 metric; (**c**) Choice R600 metric.

Based on the analysis of pedestrian network accessibility, the average value of the Angular Connectivity of children’s school travel road in the study area was 3.55, the road network density was 8308.67 m/km^2^, the intersection density was 17.96 PCs./km^2^, and the overall Connectivity was good. Influenced by topography and urban development and construction, the north side of Qiwei Road, Beichang Road, east side of Beishiwujing Road and east side of Shiwujing Road have formed road network forms such as T-junctions and broken roads, which have affected the degree of pedestrian network connectivity to a certain extent and reduced pedestrian accessibility; according to the children’s 10-min walking distance of 600 m, the degree of “Choice R600 metric” and the degree of “Integration R600 metric” were calculated. Among them, the high value of “Choice R600 metric” depended on the area with higher road network density, forming a spatial distribution characteristic of two composite corridors radiating outwards with the pedestrian space on both sides of Qiwei Road and Bawei Road as the axis. The high value of “Integration R600 metric” was centered on Qiwei Road, Bawei Road, Qijing Road and Bajing Road, forming an elliptical circle radial structure dominated by the north-south direction (Figure 9).

**Figure 10:**
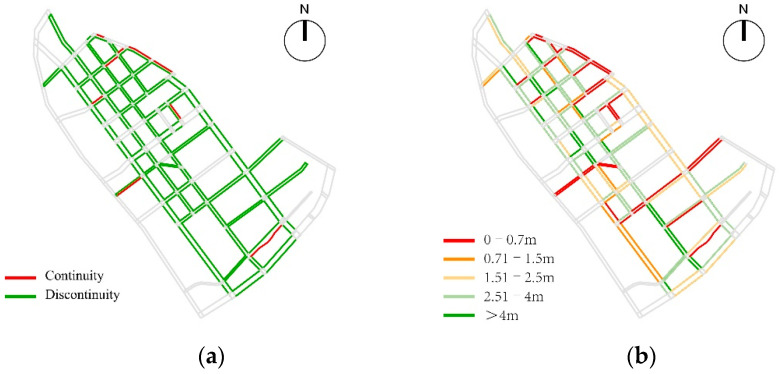
The analysis of pedestrian space accessibility: (**a**) Spatial Connectivity; (**b**) Effective Walking Width.

Based on the analysis of pedestrian space accessibility, the overall situation of pedestrian space continuity of children’s school travel road in the study area was generally good. The pedestrian fracture points were mainly concentrated in the edge area of the area, the construction area, the severely damaged section of the existing pedestrian path and the section of the unpaved pedestrian path, including the Beichang Road, the east section of Qijing Road, the west section of Bajing Road, and the west section of shierjing Road, the southern section of Shiwujing Road, Shiwei Road, and some existing sections under construction. The average effective walking width in the study area was 2.11 m, and the high values were mainly distributed in the north section of Liuwei Road, the north section of Qiwei road and Bawei road. The current sidewalk space as a whole was affected by parking, municipal facilities and commercial layout, forming a narrow pedestrian space, which greatly affected the efficiency of the use of pedestrian space and reduced the pedestrian accessibility (Figure 10).

**Figure 11:**
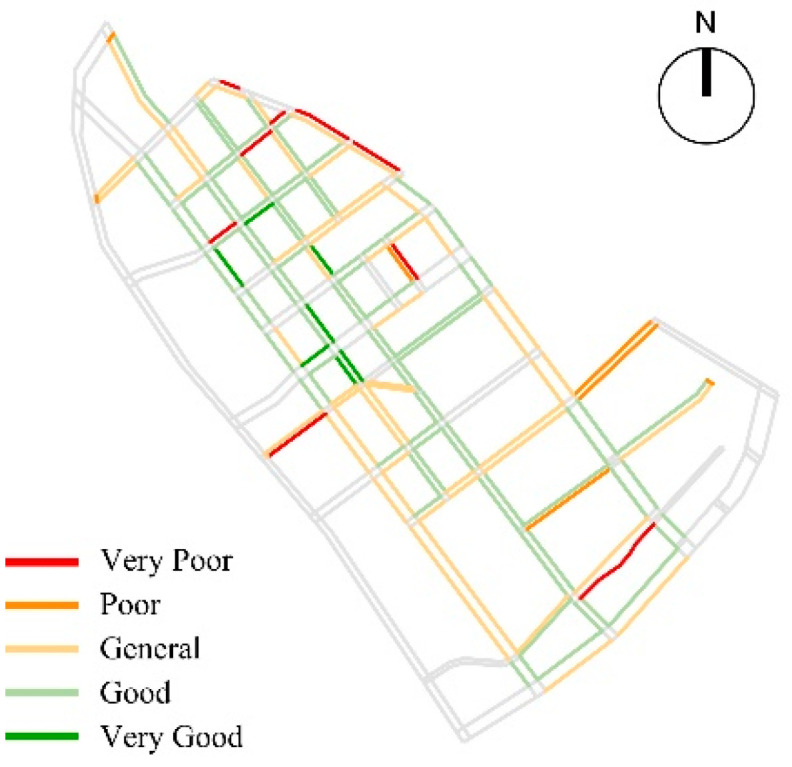
Comprehensive analysis of Pedestrian accessibility.

Based on the calculation of entropy weight method, the comprehensive evaluation result of pedestrian accessibility of children’s school travel road in the study area were obtained, and the evaluation results were spatially differentiated. According to the analysis of spatial differentiation characteristics, it was found that the high value was distributed in Liuwei Road, Qiwei Road, Bawei road and other sections, which had finger-like extensions depending on the main road. Among them, only 53.63% of the roads had good pedestrian accessibility, 34.64% of the roads had average pedestrian accessibility, and more than 11.73% of the roads had poor accessibility (Figure 11).

### 3.3. Pedestrian Safety Evaluation Based on Visibility Graph Analysis and Street View Data Analysis

**Figure 12:**
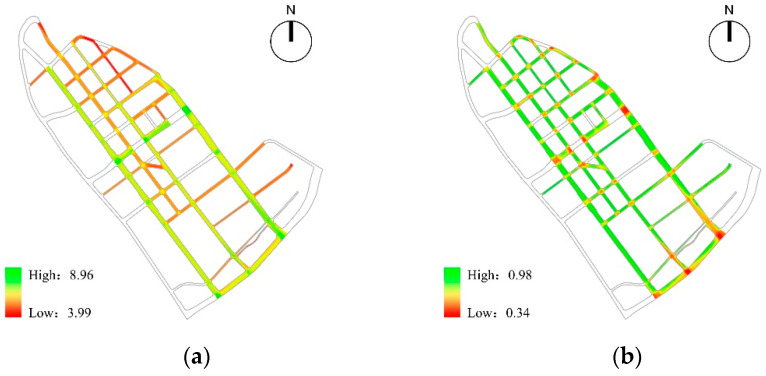
The analysis of pedestrian network safety: (**a**) Visual Integration; (**b**) Visual Clustering Coefficient.

Based on the analysis of pedestrian network security, the average value of Visual Integration of children’s school travel road in the study area was 6.38, and its high values were mainly distributed on Liuwei Road, Bawei Road, Jintang Road, Shiyijing Road and Dazhigu West Road, forming the three-axis two-zone spatial distribution characteristics; The value of the Visual Clustering Coefficient in the study area was between 0.37 and 0.97, with obvious fluctuations. Through spatial coupling analysis, it can be found that there was an obvious positive correlation between the low value of the Visual Clustering Coefficient and the road intersection. Among them, the intersections of Beichang Road-Jintang Road, Jintang Road-Shiyijing Road, Jintang Road-Shiwujing Road, and Jintang Road-Dazhigu West Road had a lower Visual Clustering Coefficient and lower degree of shielding, which promoted the occurrence of “natural surveillance” in the streets and improved the walking safety (Figure 12).

**Figure 13:**
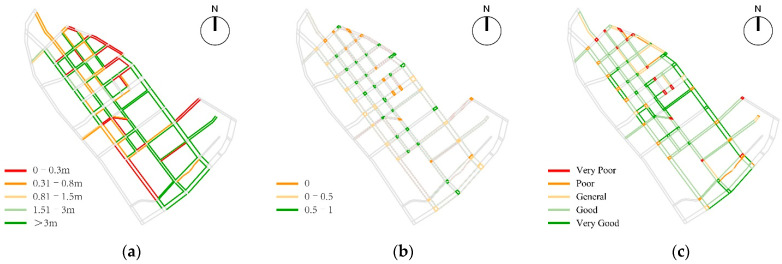
The analysis of pedestrian space safety: (**a**) the Obstruction of Pedestrian Safety; (**b**) the Completeness of Crossing Facilities; (**c**) the Impact Degree of Traffic Flow.

Based on the analysis of pedestrian space safety, the average value of the obstruction of pedestrian safety of children’s school travel road in the study area was 1.09 m, and more than 44.69% of the roads’ walking environment was affected by safety hazards such as motor vehicles, bicycle parking and unsafe municipal facilities; as for the safety of the crossing environment, the overall situation of the crossing facilities in the study area was general, with an average of 0.59. There were 14 intersections with unsafe crossing facilities, 18 intersections with insufficient crossing facilities, and only 42.86% of the intersections had complete crossing facilities; in terms of traffic environment safety, the average impact degree of traffic flow in the study area was 1.39, and the overall traffic environment was relatively smooth, with congestion on individual road sections. Among them, Bawei road was affected by three primary school students studying at the same time, resulting in extreme congestion on the road section and reducing children’s sense of walking safety (Figure 13).

**Figure 14:**
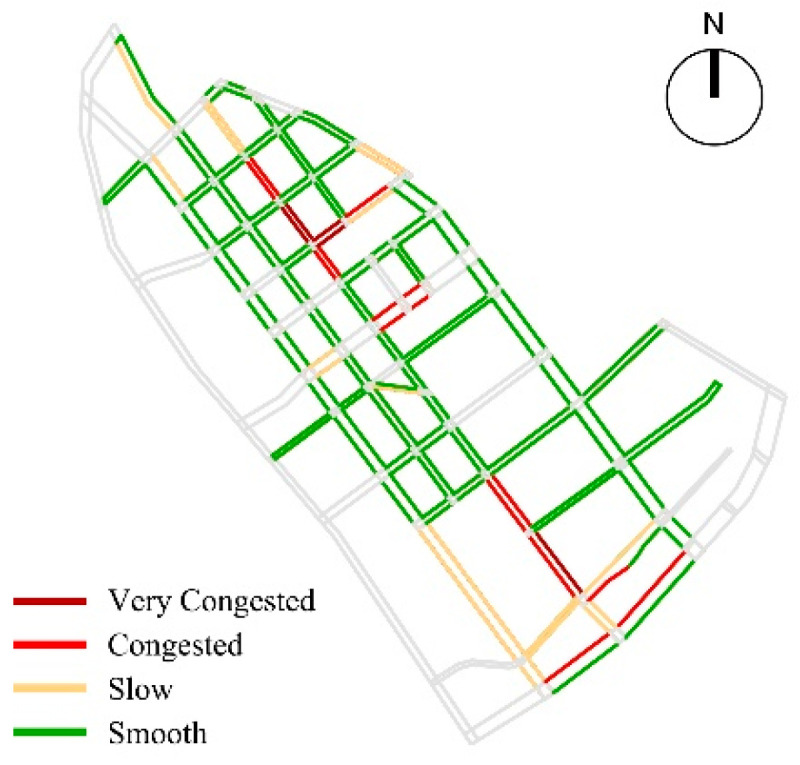
Comprehensive analysis of Pedestrian safety.

Based on the calculation of entropy weight method, the comprehensive evaluation result of pedestrian safety of children’s school travel road in the study area were obtained, and the evaluation results were spatially differentiated. According to the analysis of spatial differentiation characteristics, it was found that the lack of crossing facilities at intersections had the greatest impact on pedestrian safety, and the dense branch network was more prone to pedestrian safety hazards such as vehicle occupation and incomplete crossing facilities. Among them, only 71.91% of the roads had good pedestrian safety, 7.26% of the roads had average pedestrian safety, and more than 20.83% of the roads had poor safety (Figure 14).

### 3.4. Comprehensive Evaluation on Pedestrian Accessibility and Safety of School Travel Road

**Figure 15:**
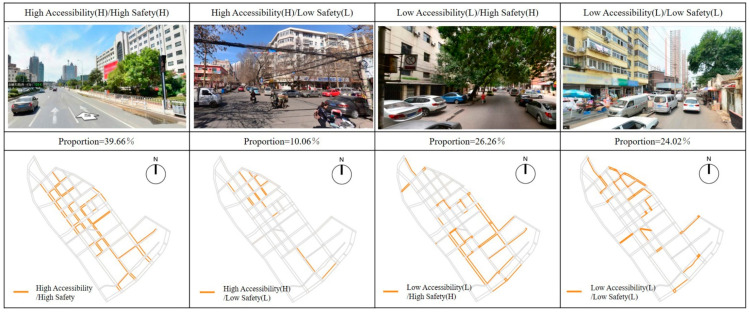
Typical Street scenes and distributions of the four types of streets.

Combined with the above results, this study carried out a superposition analysis on the walkability and safety of children’s school travel road in the study area, and evaluated the current situation of the road. The purpose is to explore the pedestrian accessibility and safety of each section of school travel road, clarify the current situation and reasons, and provide support for the regeneration and optimization of school travel road. In this superposition analysis, the accessibility was divided into “high accessibility” and “low accessibility” by taking the median value of comprehensive evaluation results of pedestrian accessibility as the boundary, and the safety was divided into “high safety” and “low safety” according to the median value of comprehensive evaluation results of pedestrian safety. The school travel roads were divided into four categories: High Accessibility (H)/High Safety (H); High Accessibility (H)/Low Safety (L); Low Accessibility (L)/High Safety (H); Low Accessibility (L)/Low Safety (L) (Figure 15).

- High Accessibility/High Safety: This kind of school travel roads had higher scores of pedestrian accessibility and pedestrian safety, and had better space potential. In the study area, such road sections were mostly concentrated in the north section of Liuwei Road, north section of Qiwei Road, south section of Bawei Road, Qijing Road, Bajing Road, east section of Shijing Road, east section of Shierjing Road, North Shiwujing road and south section of Jintang road.
- High Accessibility/Low Safety: Such road sections were mostly concentrated in the northern section of Bawei Road, the northern section of Jiuwei Road, and some sections of Liuwei Road, Qiwei Road, Qijing Road, and Bajing Road, which were generally the main roads connecting the area. The road network structure was generally accessible and had a relatively continuous walking space. However, due to the problems of motor vehicle parking and lack of pedestrian crossing facilities, it interfered with children’s pedestrian school travel behavior and had low pedestrian safety.
- Low Accessibility/High Safety: Such road sections were mostly concentrated in the road sections with higher road grades, such as the southern section of Liuwei Road, the northern section of Jintang Road, the eastern section of Shisijing Road, the Beiwujing Road, and the Shifivejing Road, etc. On the one hand, due to the influence of discontinuous sidewalk and blocked T-junction, the accessibility of pedestrian network was poor. On the other hand, due to the influence of parking occupation, municipal facilities occupation and commercial layout, the sidewalks were relatively narrow in width and poor in accessibility.
- Low Accessibility/Low Safety: Such sections were mainly concentrated in the north section of Qiwei Road, the east section of Jiujing Road, the west section of Shierjing Road, Xijin Road and Beichang Road. Affected by unreasonable road network structure, imperfect street facilities, and inadequate planning and management, this type of road needed to be improved in terms of pedestrian accessibility and safety, which needed to be focused on in the follow-up regeneration and reconstruction.

**Figure 16:**
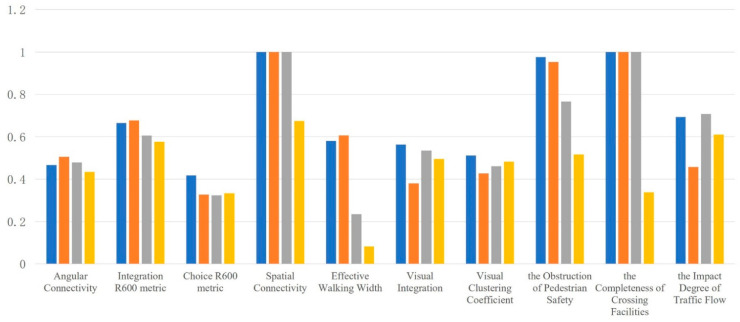
Comparative analysis of the mean values of spatial characteristics of various impact factors in the four types of roads.

By comparing the mean values of spatial characteristics of various impact factors in the four types of roads (Figure 16), it was found that for the Jintang Road area of Tianjin, the spatial characteristics of the impact factors such as the effective walking width, the spatial connectivity, the visual integration, the obstruction of pedestrian safety, the completeness of crossing facilities and the influence of traffic flow were quite different. That is to say, they had great influence on pedestrian accessibility and safety. On this basis, the objective evaluation results were compared with the on-site survey results, and the comparison found: (1) In the accessibility evaluation of school-age children and their parents, the discontinuity caused by the sidewalk being occupied and the narrow sidewalk were the important reasons for the current inaccessibility of school travel road, which was consistent with the objective evaluation results. (2) In the safety evaluation, it considered that the unreasonable road occupation of motor vehicles, bicycles and street facilities, the lack of street crossing facilities and the large traffic flow affected the safety of the current school travel road, which was highly related to the objective results, and further verified the rationality of the objective evaluation method.

## 4. Conclusions and Prospect

### 4.1. Conclusions

In the context of the construction of Children Friendly City, this study explored the accessibility and safety requirements of children in the community life circle to carry out the current evaluation and optimization research of the walkability control index of school travel road on the block scale and the human scale and provided support for subsequent planning and management by constructing a research path of “Status Evaluation-Optimization Strategy-Post Optimization Evaluation”. The research conclusions are as follows: (1) Based on the multi-source data of space, time and attribute, this paper evaluated the pedestrian accessibility and safety of children’s school travel road and found that the pedestrian accessibility and safety of the current school travel road need to be improved, and only 39.66% of the roads with high accessibility/safety. (2) By comparing the average spatial characteristics of various impact factors in the four types of roads, it was found that the main impact factors of the pedestrian accessibility and safety of the school travel road were the effective walking width, the spatial connectivity, the visual integration, the obstruction of pedestrian safety, the completeness of crossing facilities and the influence of traffic flow. (3) To sum up, it is concluded that in the process of street regeneration in the future, we should focus on solving the problems of unreasonable road network structure, occupied pedestrian space, imperfect street facilities, and inadequate planning and management, and then put forward effective regeneration and optimization scheme from the perspective of planning, design and management.

### 4.2. Optimization Strategy

Based on the above analysis, this study compared the evaluation results with the optimization strategies and positive examples of commuting roads in the street design guidelines of Beijing and Shanghai, put forward the space optimization strategies from the three aspects of “network-space-node”, and put forward the planning management strategies from the perspective of urban governance.

#### 4.2.1. Pedestrian Network: Optimize the Pedestrian Network of School Travel Road to Meet the Requirements of Pedestrian Accessibility

This study put forward the regeneration strategies of optimizing the pedestrian network, such as optimizing the road line, merging the nearest T-junction as the intersection, and opening the dead-end road, so as to meet the requirements of pedestrian accessibility. After the optimization of the pedestrian network, a total of 14 intersections have been dredged and improved, two roads have been opened, and there are nine more roads for children to walk to school than the current situation. Among them, the “Integration R600 metric” increased from the current average 86.90 to 92.45; the “Choice R600 metric” increased from the current average 2.04 to 2.15.

#### 4.2.2. Pedestrian Space: Intensive Use of Street Space to Achieve Spatial Integration of School Travel Road

**Figure 17:**
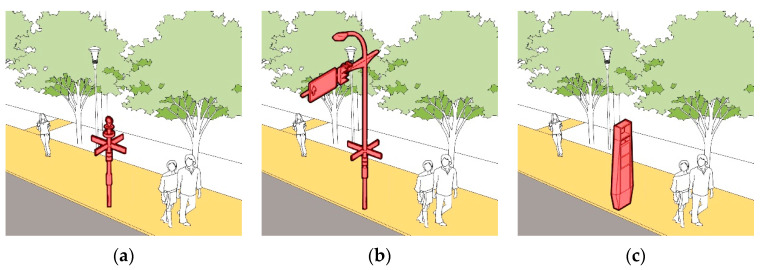
Schematic Design of Multi Pole Integration: (**a**) Street Lamp Pole as the Basis of Integration; (**b**) Traffic Pole as the Basis of Integration; (**c**) Information Board Integration.

**Figure 18:**
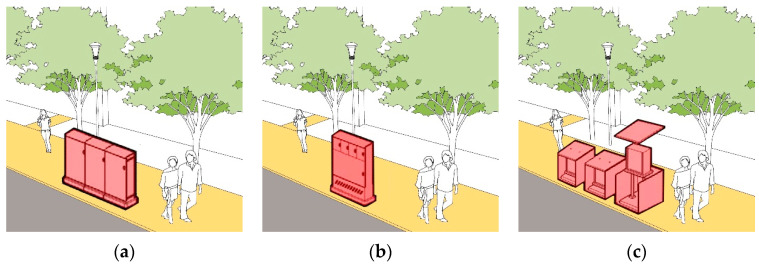
Schematic Design of Multi Box Union: (**a**) Box integrated layout; (**b**) Multi box in one; (**c**) Box blanking.

Through the combination of multiple boxes, the integration of multiple poles and the entry of overhead lines into the ground, all kinds of municipal boxes that affect walking safety will be gradually moved indoors or underground; through the integration of green belts, facility belts, isolation belts and other spaces, the compound utilization of pedestrian space is realized (Figure 17 and Figure 18). After the optimization, “Obstruction of Pedestrian Safety” decreased from the current average value of 1.09 to 0.53. Additionally, the “Effective Walking Width” was increased from the average 2.11 m to 2.95 m.

#### 4.2.3. Important Nodes: Refined Design of Intersection and School Front Space to Achieve Traffic Calm and Stability

**Figure 19:**
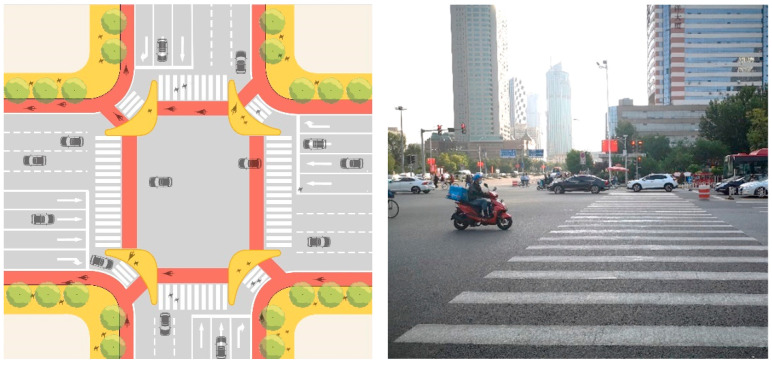
Right Transductive Island of Intersection between Xijing Road and Liuwei Road.

**Figure 20:**
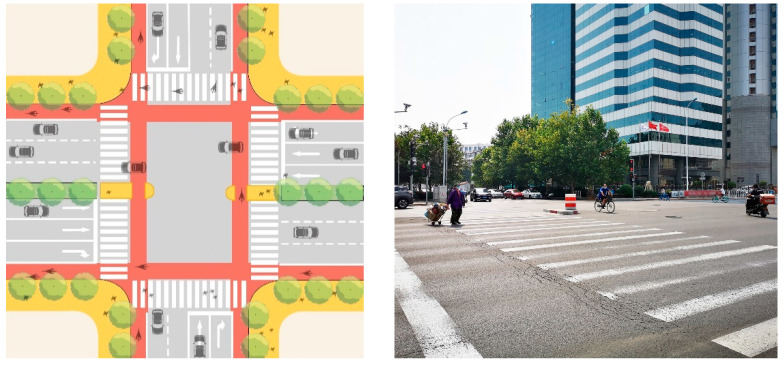
Setting Up a Safety Island at the Intersection of Shiyijing Road and Bawei Road.

This study proposes to reasonably planning of traffic flow around the school, and encourage the static stability design of the traffic around the school, so as to enhance the walking experience and enhance the safety of walking through school (Figure 19 and Figure 20). After optimization, the completeness of crossing facilities increased from the current average 0.59 to 1, an increase of 69.49%.

#### 4.2.4. Fine Governance: Coordinate Traffic Management, and Implement Traffic Control on Special Sections during School Hours

**Figure 21:**
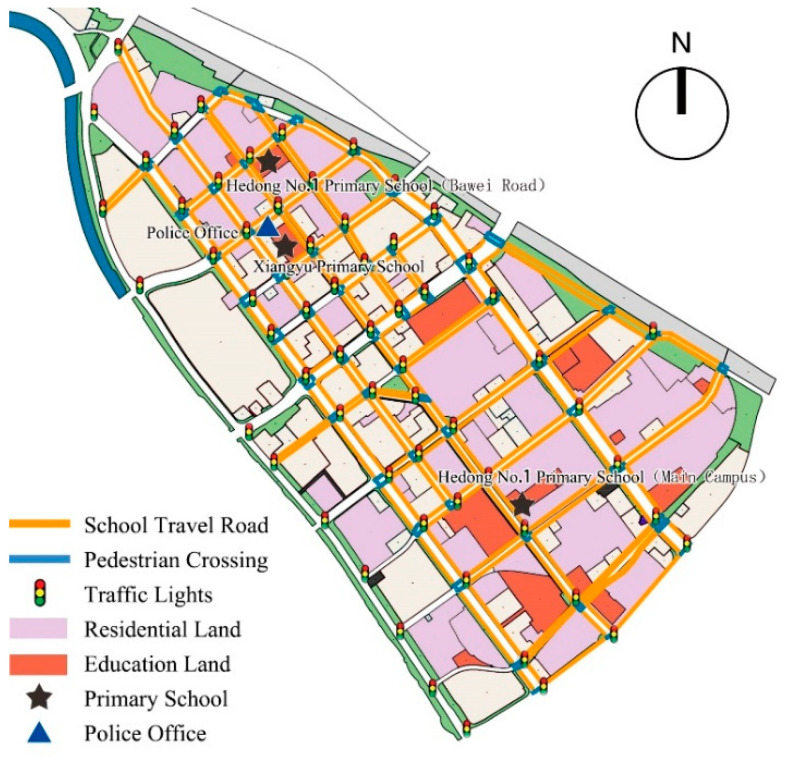
School Travel Routes Planning of the Study Area.

It is proposed to coordinate traffic management and implement traffic control (speed limit or ban) on the general school roads around the school. At the same time, it also puts forward the proposal map of school travel routes, and mark the important infrastructures and potential risk points on the school travel roads, so as to strengthen the children’s awareness of road traffic safety (Figure 21). After optimization, the “the impact degree of traffic flow” decreased from the average value of 1.39 to 1.28, and the influence of traffic factors on children’s pedestrian school travel was improved.

### 4.3. Simulation Verification

**Figure 22:**
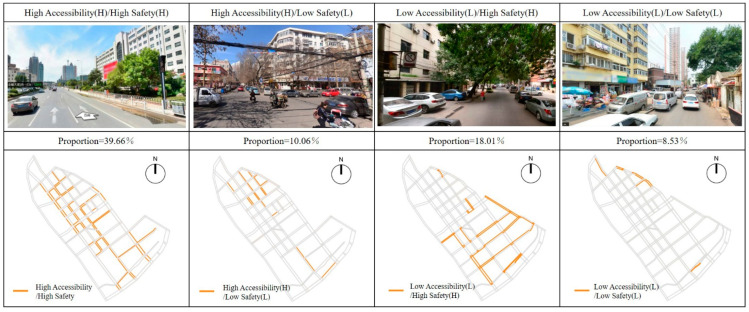
Typical Street scenes and distributions of the four types of streets after optimization.

Combined with the optimization strategy and the change of related parameters, the optimized children’s school travel road regeneration plan was substituted into the above evaluation model for simulation verification. The evaluation results are shown in Figure 22: After the optimization, the pedestrian accessibility and safety of the children’s school travel roads in the study area have been significantly improved. Among them: High Accessibility (H)/High Safety (H) roads accounted for 66.35%, increased by 67.30%(39.66%) compared with the current situation; High Accessibility (H)/Low Safety (L) roads accounted for 7.11%, with a decrease of 29.32% (10.06%) compared with the current situation; Low Accessibility (L)/High Safety (H) roads accounted for 18.01%, with a decrease of 31.42% (26.26%) compared with the current situation; and the Low Accessibility (L)/Low Safety (L) roads accounted for 8.53%, a decrease of 64.57% (24.02%) from the current situation. In conclusion, the children’s school travel road in Jintang Road area has been effectively improved.

### 4.4. Research Prospect

This paper studies the part of the “control index” of children’s walkability on the school travel road. In the follow-up, further research on “guidance indicators” will be carried out to meet the comfort needs of children’s school travel road, and to provide more flexible guidance for the optimization of children’s pedestrian school travel roads. At the same time, the combination of “guidance index” and “control index” will jointly optimize and supplement the pedestrian-friendly street design guidelines. By integrating relevant standards of different professions, the existing contents are supplemented, optimized and refined, and optimized strategies and effective guidelines are proposed from the perspectives of general principles of school travel road design, spatial design guidelines, element design guidelines, and management implementation strategies. With the introduction of the fourth generation of urban design paradigm—digital urban design based on human-computer interaction [39], the use of intelligent management and monitoring platform can be effectively applied to the construction of children’s school travel road. Through the construction of monitoring and early warning modules such as online simulation and traffic prediction, intelligent identification and early warning of road danger, and risk map of pedestrian school travel road, it can provide monitoring, guarantee and early warning information for children’s school travel road [40].

## Figures and Tables

**Table 1:** Pedestrian accessibility and safety index system of children’s school travel road.

First-Level Indicators	Secondary Indicators	Third-Level Indicators	Calculation Formula	Quantitative Interpretation
**Accessibility**	Pedestrian Network Accessibility	Angular Connectivity	$C_{i}=k$	The spatial syntax “angular connectivity” index is used to quantify. *k* refers to the number of other school travel roads directly connected to a school travel road.
		Choice R600 metric	$C_{i}=\frac{Choice_{i}}{\;Total\;depth_{i}}$	Combined with children’s 10-min walking distance of 600 m, the ratio of the spatial syntax “Choice (R = 600)” to “Total depth (R = 600)” is used to quantify.
		Integration R600 metric	$I_{i}=\frac{D_{k}\left(k-2\right)}{2\left(MD-1\right)}$	Combined with children’s 10 min walking distance of 600 m, the space syntax “integration (r = 600)” is used to quantify. *MD* represents the average depth, *D_k_* represents the symmetry of diamond structure, and *k* represents the number of elements of diamond structure
	Pedestrian Space Accessibility	Spatial Connectivity	$S_{i}=\frac{L_{sidewalk}\;}{L_{road}}$	It indicates the continuity of sidewalk, which is quantified by the ratio of the length of sidewalk within the road section to the length of the whole road.
		Effective Walking Width	$W_{i}=\alpha *W_{total\;width}-W_{occupied\;width}$	It represents the actual width of the sidewalk in the process of school travel, and is quantified by the difference between the total width and the occupied width after subtracting the facility zone.
**Safety**	Pedestrian Network Safety	Visual Integration	$V_{i}=\frac{1}{RRA_{i}}=\frac{D_{k}}{RA_{i}}$	The spatial syntax “Visual Integration” index is used to quantify. *V_i_* is the Visual Integration of node *i*; *RRA_i_* is the true relative asymmetry, *RA_i_* is the relative asymmetry, *k* is the number of spatial units, and *D_k_* is the standard value of *RRA_i_* when standardizing *RA_i_*.
		Visual Clustering Coefficient	$V_{i}=\frac{K}{k\left(k-1\right)}$	The spatial syntax “Visual Clustering Coefficient” index is used to quantify. *K_i_* represents the line of sight aggregation coefficient in the spatial area of the general road, *k*_1_ represents the number of nodes with line of sight depth of 1, and *k*_2_ represents the number of nodes with line of sight depth of 2.
	Pedestrian Space Safety	Safety of Walking Environment	$Z_{i}=\frac{\sum_{i=1}^{n}H_{Obstruction}}{n}$	It indicates the safety of sidewalk space during pedestrian school travel, which cannot be directly quantified. To a large extent, it is affected by the occupation of sidewalks, which is quantified by “Obstruction of Pedestrian Safety”. *Z_i_* represents the average pedestrian safety obstruction of a school travel road section, *H_Obstruction_* represents the road occupation width of various permanent and temporary pedestrian safety obstacles in the school travel road of a sampling point, and n represents the number of sampling points in a school travel road section.
		Safety of Crossing Environment	Complete Facilities = 1; Insufficient Facilities = 0.5; No Facilities = 0	It indicates the safety of the intersection space during pedestrian school travel, and quantifies it with the “Completeness of Crossing Facilities” of the intersection.
		Safety of Traffic Environment	Smooth = 1; Slow = 2; Congestion = 3; Extreme Congestion = 4	It indicates the impact of the traffic environment such as vehicles on the safety of children’s pedestrian school travel, and is quantified by “Impact Degree of Traffic Flow”.

Source: drawn by the author according to references [33,34,35,36,37,38].

**Table 2:** Weights of pedestrian accessibility indicators of children’s school travel roads.

First-Level Indicators	Secondary Indicators	Third-Level Indicators	Indicator Weight
Accessibility	Pedestrian Network Accessibility	Angular Connectivity	0.03
		Choice R600 metric	0.04
		Integration R600 metric	0.11
	Pedestrian Space Accessibility	Spatial Connectivity	0.34
		Effective Walking Width	0.49

Source: by the author.

**Table 3:** Weights of pedestrian safety indicators of children’s school travel roads.

First-Level Indicators	Secondary Indicators	Third-Level Indicators	Indicator Weight
Safety	Pedestrian Network Safety Pedestrian Space Safety	Visual Integration	0.22
		Visual Clustering Coefficient	0.15
		Obstruction of Pedestrian Safety	0.07
	Pedestrian Space Accessibility	Completeness of Crossing Facilities	0.45
		Impact Degree of Traffic Flow	0.11

Source: by the author.

**Table 4:** Analysis of characteristics of children’s school travel activities and walking environment demand.

Behavior Classification	Specific Behavior	Walking Environment Needs
Walking	Walking Through	Continuous, accessible and safe sidewalk space
	Crossing the Street	Safe crossing environment and facilities guarantee
	Chasing Each Other	Safe and relatively wide walking space
Staying	Waiting	Safe waiting space
	Chatting	Enough space to stay
	Shopping	Rich street business
Playing	Playing and Running	Rich street public space and street sketches

Source of information: according to the field investigation.

## Data Availability

The data presented in this study are available on request from the corresponding author. The data are not publicly available due to confidentiality.
